# One step ahead: mapping the Italian and German cybersecurity laws against the proposal for a NIS2 directive

**DOI:** 10.1365/s43439-022-00058-7

**Published:** 2022-07-20

**Authors:** Sandra Schmitz-Berndt, Pier Giorgio Chiara

**Affiliations:** 1 University of Luxembourg, Esch-sur-Alzette, Luxembourg; 2 University of Bologna, Bologna, Italy

**Keywords:** NIS Directive, Cybersecurity, Italy, Germany, NIS2 Proposal

## Abstract

With the COVID-19 pandemic accelerating digital transformation of the Single Market, the European Commission also speeded up the review of the first piece of European Union (EU)-wide cybersecurity legislation, the NIS Directive. Originally foreseen for May 2021, the Commission presented the review as early as December 2020 together with a Proposal for a NIS2 Directive. Almost in parallel, some Member States strengthened (or adopted) national laws beyond the scope of the NIS Directive to respond adequately to the fast-paced digital threat landscape. Against this backdrop, the article investigates the national interventions in the field of cybersecurity recently adopted by Italy and Germany. In order to identify similarities and divergences of the Italian and German national frameworks with the European Commission’s Proposal for a NIS2 Directive, the analysis will focus on selected aspects extrapolated from the Commission Proposal, namely: i) the enlarged scope; ii) detailed cybersecurity risk-management measures; iii) more stringent supervisory measures; and, iv) stricter enforcement requirements, including harmonised sanctions across the EU. The article concludes that the national cybersecurity legal frameworks under scrutiny already match the core of the proposed changes envisaged by the NIS2 Proposal.

## Introduction

Mapping a comprehensive outline of dynamically evolving threats is not an easy task. The European Union Agency for Cybersecurity (ENISA) annually prepares a report on the status of European Union (EU) cybersecurity, which identifies major threats including the threat actors and attack techniques as well as describing mitigation measures. The constantly improving methodology of ENISA’s analysis^1^ reflects the changing nature of the threat landscape: cyberattacks have significantly increased through the years 2020 and 2021 not only in terms of vectors and numbers but also in terms of their impact and sophistication, with the COVID-19 pandemic contributing to an increased attack surface [8]. Despite a growing awareness among different actors—individuals, businesses, public bodies, institutions, organisations—about their vulnerabilities to cyber threats [23], appropriate guidelines, training and procedures are still scarce [9, 21].

Already on 16 December 2020, the European Commission presented the new EU Cybersecurity Strategy [15]—a key, integrated component of the European Digital Transition Plan [13], the Recovery Plan [14] and the European Security Strategy [10], with the aim of leading the efforts for secure digitalisation. The Strategy deploys three principal instruments to address three areas of EU action: i) resilience, technological sovereignty and leadership; ii) building operational capacity to prevent, deter and respond; and, iii) advancing a global and open cyberspace.

The ambitious and challenging goal of strengthening and enhancing the Union’s cybersecurity is further substantiated by two legislative proposals: the Proposal for a NIS2 Directive [11] and a new Directive on Critical Entity Resilience (CER) [12]^2^. For the purpose of this work, the focus will be solely on the European Commission Proposal for a NIS2 Directive (NIS2 Proposal)^3^, which will replace the NIS Directive.^4^

At the same time, national legislators have also been actively seeking solutions to respond to the increased cybersecurity threats landscape. Italy, through the establishment of the *Perimetro di Sicurezza Nazionale Cibernetica*, decided to further strengthen its rules and procedures on network and information systems (NIS) in order to ensure a higher level of security of the NIS of public administrations, as well as national public and private entities and operators that are relevant for the national security.

In May 2021, Germany passed the *Zweites Gesetz zur Erhöhung der Sicherheit informationstechnischer Systeme *(ITSiG 2.0), which significantly amended the pre-existing national cybersecurity law by extending inter alia the scope of the central German Cybersecurity Act and tightening NIS security obligations.

Since the NIS2 Proposal remains a Directive, this article aims at assessing the maturity of the existing Italian and German national cybersecurity legal frameworks against the foreseen NIS2 legal standard. In particular, the analysis aims to identify similarities as well as divergences of the existing national frameworks with the NIS2 Proposal.

The remainder of the article is organised as follows: Sect.2 identifies four major changes to the status quo suggested by the NIS2 Proposal that shall serve as a guide in the analysis of the national legal acts. In the following, Sects. 3 and 4 assess the main procedural and substantial aspects of the Italian and German cybersecurity regime against the benchmark of the NIS2 Proposal. Finally, Sect. 5 draws some conclusions as regards the level of maturity of the two national regimes against the background of the NIS2 Proposal and comments on the rush forward by national legislators.

## The proposal for a NIS2 Directive

Although the first review of the NIS Directive (NISD) was originally foreseen for completion in May 2021, the European Commission published a Proposal for a NIS2 Directive as early as December 2020. The Proposal seeks to modernise the existing legal framework and addresses several weaknesses that prevented the existing Directive to unlock its full potential. Among the systemic and structural changes envisaged by the NIS2 Proposal^5^, this paper identifies four key changes to the NISD, which serve to outline existing deficiencies and responses to these. These four main thematic areas are: i) the enlarged scope of the NISD; ii) revised cybersecurity risk management measures and reporting duties; iii) more stringent supervisory powers; and iv) the introduction of harmonised administrative sanctions. These four regulatory drives that underpin the revision of the NISD are addressed in the following and will subsequently serve as benchmarks against which the national regimes will be matched.

### The scope of the proposal for a NIS2 Directive

The first key change concerns the scope of the NIS2 Proposal. The explanatory memorandum to the Proposal [11] acknowledges that the increased digitisation of recent years and the higher rate of interconnectedness are crucial factors contributing to the gradual inadequacy of an overly limited scope of the NISD. The NISD no longer succeeds in reflecting all digitised sectors that provide key services in the Union [11]. As a consequence, not only does the NIS2 Proposal introduce an enlarged definition of what is seen as critical infrastructures, but also the distinction between operator of essential services (OESs) and digital service providers (DSPs) is replaced by differentiating between *essential entities (EEs)* and *important entities (IEs).*^6^ This structural change is based on the assumption that the differentiation between OESs and DSPs does not reflect the actual importance of the sectors or services for the internal market [11]. In contrast, the new classification of EEs and IEs takes into account the level of criticality of the sector or of the type of service provided, as well as the level of dependency of other sectors/services. Accordingly, the more critical EEs operate in the sectors listed in Annex I NIS2 Proposal, which include those entities that are considered an OES under the NISD: energy; transport; banking; financial market infrastructures; health; drinking water; digital infrastructure. The Proposal further suggests re-including the sectors waste water, public administration^7^ and space.^8^ IEs operate in the sectors listed in Annex II NIS2 Proposal and include the previously non-encompassed sectors postal and courier services; waste management; manufacture, production and distribution of chemicals; food production, processing and distribution; manufacturing^9^ and digital providers^10^.

Whereas under the NISD competent authorities had to identify OESs on a national basis based on national criteria, the NIS2 Proposal foresees a uniform criterion in form of a size-cap rule across the Union to determine the entities falling within the scope of application of the Directive.^11^ Recognising that the size-cap rule may not be appropriate for all services in all Member States, Article 2(2) NIS2 Proposal enlists exceptions for which the Directive applies to entities regardless of their size.

### Cybersecurity risk management and incident reporting obligations

The NISD has already introduced security and incident reporting obligations. These obligations slightly vary depending on whether the entity concerned is an OES or DSP, for instance in the sense that OESs have to report incidents having a significant impact on the continuity of the essential services while DSPs have to report incidents having a substantial impact on the service provided. However, this is only a minor blur compared to the discretion that was provided to the Member States as regards the implementation of the security and incident reporting obligations. The wide discretion resulted in significantly different national implementation. In order to achieve a more harmonised approach, the NIS2 Proposal explicitly includes (technical) cybersecurity management measures or controls and strengthens incident notification obligations.^12^ Further, the provisions on security measures (Article 18) and reporting obligations (Article 20) no longer differentiate between the entities concerned.

Article 20 NIS2 Proposal requires Member States to ensure that EEs and IEs notify the competent authorities or the Computer Security Incident Response Teams (CSIRTs) without undue delay, and in any event within 24 h^13^ after having become aware of the incident having a significant impact on the provision of their services. In contrast to the NISD which defined an incident as ‘any event having an actual adverse effect on the security of network and information systems’, Article 4(5) NIS2 Proposal provides a more sophisticated definition setting forth that an incident means “any event compromising the availability, authenticity, integrity or confidentiality of stored, transmitted or processed data or of the related services offered by, or accessible via, network and information systems”. Notably, reporting is no longer restricted to incidents with a substantial or significant impact, but also encompasses incidents that have the potential to cause “substantial operational disruption or financial loss” or have the potential to cause “considerable material or non-material losses”.^14^ This means that an incident is considered significant even if the incident only has the potential to cause harm, but the harm must not have materialised. Further, the new provision partially diverges from the “without undue delay” standard of the NISD by requiring notification within 24 h. Article 20(2) NIS2 Proposal extends reporting to significant “cyber threats” that could have potentially resulted in a significant incident.^15^ In that regard, Recital 24 NIS2 Proposal specifies that the additional information should aid Member States to adapt their level of preparedness and be adequately equipped “to prevent, detect, respond to and mitigate network and information incidents and risks”.

In order to acquire a full picture of the threat landscape, Article 27 NIS2 Proposal provides a legal basis for voluntary notifications of significant incidents, cyber threats and near misses by entities *falling outside* the scope of the NIS2 Directive. Member States may prioritise the processing of mandatory notifications over voluntary notifications.

In terms of cybersecurity management measures, Article 18(2) NIS2 Proposal details a minimum list of cybersecurity measures that entities have to take to manage the risks posed to their NIS. These measures include: (i) risk analysis and information system security policies; (ii) incident handling (prevention, detection and response to incidents); (iii) business continuity and crisis management; (iv) supply chain security; (v) security in network and information systems acquisition, development and maintenance, including vulnerability handling and disclosure; (vi) testing and auditing; and (vii) the use of cryptography and encryption. Notably, the NIS2 Proposal addresses, for the first time, cybersecurity of the information and communications technology (ICT) supply chain, which is of special importance in the case of the Internet of Things (IoT), but also responds to incidents, where malicious actors compromise the security of an entity’s NIS by exploiting vulnerabilities affecting third party products and services.^16^ Supply chain security includes security-related aspects concerning the relationship between an entity and its suppliers or service providers. To further address key supply chain risks and assist entities covered by the Directive to appropriately manage supply chain and supplier related cybersecurity risks, Article 19 NIS2 Proposal introduces coordinated supply chain risk assessments replicating Recommendation (EU) 2019/534 on Cybersecurity of 5G networks^17^. The supply chain risk assessment should also take into account non-technical factors including those defined in the aforementioned Recommendation.^18^

### Supervision

Although the NISD required Member States to ensure that the competent authorities have the necessary powers and means to assess the compliance with the security and notification requirements, the supervision and enforcement regime of the NISD has proven ineffective [11]. Accordingly, the NIS2 Proposal seeks to strengthen supervisory powers via a minimum list of actions and means by which competent authorities may ensure effective compliance. While EEs will be subject to a fully-fledged supervisory regime, a light supervisory regime, that is, *ex-post* only, will apply to IEs^19^, mirroring the so-called ‘light-touch’ approach applied to DSPs under the NISD^20^. Pursuant to Article 29(2) NIS2 Proposal, the new measures include, inter alia: on-site inspections and off-site supervision, random checks as well as regular audits, requests for evidence of implementation of cybersecurity policies, such as the results of security audits carried.

### Enforcement and sanctions

As mentioned above, the enforcement regime has proven ineffective, although Article 21 NISD required Member States to introduce a penalty regime with effective, proportionate and dissuasive penalties. In practice, Member States have been reluctant to apply penalties for failure to comply with the security or incident notification requirements [11]. In order to strengthen the enforcement regime, Article 31 NIS2 Proposal lays down a minimum list of administrative sanctions for breach of the cybersecurity risk management and reporting obligations. Mirroring the sanctioning scheme of Article 83(4) GDPR, Article 31(4) NIS2 Proposal foresees severe administrative fines of up to €10 M or 2% of the total worldwide annual turnover of the undertaking to which the entity belongs to in the preceding financial year, whichever is higher.^21^ The NIS2 Proposal further introduces a form of ‘managerial liability’: based on a proportionality criterion, and eventually as *extrema ratio*^22^, Article 29(5)(b) NIS2 Proposal provides for Member States to impose a temporary ban against any person discharging managerial responsibilities at chief executive officer or legal representative level in that essential entity, and of any other natural person held responsible for the breach, from exercising managerial functions in that entity. Sievers [20] interprets this provision as a “piercing of the corporate veil”. Nevertheless, it can be argued that such accessory administrative sanction finds its rationale in the potentially devastating impact of cyber-incidents on entities’ activities—and ultimately on their consumers—stemming from the infringement of legal requirements. Given the severe character of such sanctions, Recital 76 NIS2 Proposal considers that “they should only be applied proportionally to the severity of the infringement and taking account of the specific circumstances of each case, including the intentional or negligent character of the infringement, actions taken to prevent or mitigate the damage and/or losses suffered”.

## The Italian cybersecurity legal framework

**Fig. 1:**
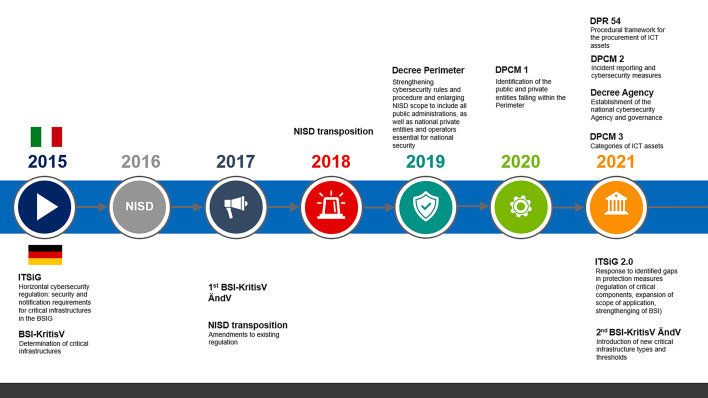
Timeline of the evolution of the Italian and German cybersecurity legal frameworks

The NISD has been transposed into the Italian legal system by *Decreto Legislativo* no. 65 of 18 May 2018^23^, which sets out the legislative framework for the NIS security measures to be adopted and identifies the competent actors to implement the obligations laid down by the EU cybersecurity legal framework (Fig. 1).

However, the Italian government decided to strengthen rules and procedures with a view to ensuring a higher level of security of networks, information systems and IT services of public administrations, as well as of national public and private entities and operators, through the establishment of the so-called “national cybersecurity perimeter” (*Perimetro di Sicurezza Nazionale Cibernetica*) by means of *Decreto-Legge *(Decree Law) of 21 September 2019 (hereinafter, Decree Perimeter)^24^ (Fig. 1).

The rationale underlying the adoption of the Decree Perimeter is the establishment of a coherent and comprehensive legal framework that enhances the scope of the NISD [4] to uphold national security. Indeed, the limited scope of the NISD does not fully cover the totality of public and private operators on which an *essential functioning of* the State or the *provision of an essential service* for the maintenance of civil, social or economic activities fundamental in the interests of the State depend; the malfunctioning, interruption or improper use of these services may however be detrimental to national security.^25^

Against this background, Article 1(8) of the Decree Perimeter links foresees that OESs and DSPs observe the cybersecurity requirements outlined in the national act implementing the NISD, i.e. the *Decreto Legislativo* no. 65 of 18 May 2018, *if they are at least equivalent *to those laid down by the Decree implementing the Perimeter^26^. The national Agency for Cybersecurity is empowered by the same article to define *additional *measures in order to meet the standard of security set forth by the Perimeter.

The Decree Perimeter foresees that the implementing rules to further specify the obligations of the entities encompassed are to be defined through the adoption of three D.P.C.M. (Prime Ministerial Decree), one D.P.R. (Presidential Decree), as well as a series of acts, communications and determinations of various committees. In the following, the Italian government adopted the D.P.C.M. 30 July 2020, no. 131 (hereinafter, DPCM 1)^27^, which identifies the public and private entities falling within the Perimeter as well as the criteria for creating lists of the entities’ relevant networks, information systems and computer services^28^ (Fig. 1). Subsequently, the D.P.C.M. 14 April 2021, no. 81 (hereinafter, DPCM 2)^29^ defines the procedure for incident reporting, as well as mandatory technical security measures. Finally, the D.P.R. 5 February 2021 no. 54 (hereinafter DPR 54)^30^ lays down a procedural framework for the procurement of ICT goods to be used on networks, information systems and IT services by the entities under the scope of the Perimeter; the categories of these assets are further identified by the D.P.C.M. 15 June 2021 (hereinafter DPCM 3)^31^ (Fig. 1).

Although not originally foreseen by the Decree Perimeter, Decree-Law 14 June 2021 no. 82^32^ significantly reshapes the normative architecture of the Perimeter since it establishes the National Agency for Cybersecurity, which also hosts the national CSIRT and the National Centre for Certification and Evaluation^33^ (in Italian, ‘CVCN’, which acts as ‘national cybersecurity certification authority’ for the purpose of complying with rules set out in the Cybersecurity Act (CSA)^34^). To complete the regulatory framework envisaged by the Perimeter, the fourth DPCM establishing a network of public-private laboratories in order to support the CVCN for technological assessment constitutes the final piece of the jigsaw. The fourth DPCM is expected to be published in the Official Journal of the Italian Republic by the end of summer 2022.

### The scope of the perimeter

After the enactment of the Perimeter, the first implementing decree, the DPCM 1, entered into force on 5 November 2020. The DPCM 1 lays down the procedural criteria according to which the competent public administration will have to identify the entities encompassed by the Perimeter and the criteria that such entities must follow in the setting up and updating of the lists of networks, information systems and IT services.

The identification of the entities included in the Perimeter, performed by the public administrations per each sector of competence, follows a *risk-based *and *scalable *approach^35^. Based on a “gradual mechanism” and on a risk assessment^36^, priority has been given to the identification of the subjects operating in the governmental sector^37^, with the competent authority being the “interministerial committee for cybersecurity”^38^ established in the Presidency of the Council of Ministers^39^. Further sectors include: interior; defence; space and aerospace; energy; telecommunications; economy and finance; transport; digital services; critical technologies; social security institutions and labour.^40^

Interestingly, the list of the entities in the Perimeter shall be included in an administrative act, adopted by the President of the Council of Ministers, which, eventually, is not subject to publication.^41^ The rationale behind the non-disclosure lies in the underlying purpose of protecting national security; however, the secrecy is more formal than real, as the majority of the entities that fall in the Perimeter’s scope can be easily identified by anyone with experience in the field^42^.

The Italian legislator did not substantiate the exact content of the “digital services” sector, unlike in the case of “critical technologies”^43^, for which reference is made to Article 4(1)(b) of Regulation (EU) 2019/452^44^ as to include artificial intelligence, robotics, semiconductors, cybersecurity, aerospace, defence, energy storage, quantum and nuclear technologies as well as nanotechnologies and biotechnologies. The resulting legal uncertainty may lead to either a broad or restricted interpretation, with relevant consequences for the entities involved in terms of compliance costs if a broad understanding of “digital services” should be adopted; conversely, should a narrower interpretation of “digital services” prevail, national (cyber)security may be jeopardised as important entities may fall outside the scope of the Perimeter.^45^

Finally, a combined reading of Article 1(5) Decree Perimeter and Article 3(1) DPCM 1 provides for an element of ‘flexibility’ in terms of adjustments to the national cybersecurity legal framework. Whilst Article 1(2) and (3) Decree Perimeter lays down a legal basis for updating the implementing decrees DPCM 1 and DPCM 2, the DPCM 1 explicitly envisages a possible extension of the scope to other sectors when updating the decree.

### Cybersecurity risk management and reporting obligations

The entities falling in the Perimeter scope are obliged to prepare a list, updated on an annual basis, of the networks, information systems and IT services that make up the ICT assets under their control.^46^ Criteria and procedures are laid down in Article 7: following a scalable and risk-based approach, in accordance with the principle of graduality, those ICT assets are to be identified first that, in the event of an incident, would cause complete disruption of the essential function or service.^47^ The entities encompassed shall also describe the architecture and component parts^48^ of the ICT assets previously identified, based on a model provided by the national Cybersecurity Agency^49^. This obligation may prove to be particularly challenging, especially considering the high digitalisation rate of many operators. These lists are to be transmitted to the Agency within six months of receipt of the notice of registration in the Perimeter^50^.

Whereas a specific organisational requirement in terms of listing ICT assets and specifying their components is omitted in the NIS2 Proposal, the reporting obligations procedure and the cybersecurity risk-management measures detailed by DPCM 2 largely overlap with the provisions of the NIS2 Proposal.

Cybersecurity incidents^51^ are categorised according to their impact on ICT assets. The taxonomy of DPCM 2 makes a first binary distinction based on the gravity of an incident: Table 1 in Annex A contains less serious incidents (i.e. initial exploitation, fault, privilege escalation, defence evasion, persistence, command and control, discovery, credential access, lateral movement, collection and exfiltration) and Table 2 the more serious ones (i.e. inhibit response function, impair process control, failure). This classification is functional to the different timing needed for an effective response.^52^ Thus, the Perimeter entities shall report to the Italian CSIRT^53^ within one hour in the case of an incident identified in Table 2, Annex A, and six hours in the case of an incident covered by Table 1^54^. Those deadlines shall commence from the moment the entity becomes aware of the incident, e.g. through the monitoring, testing and control activities carried out on the basis of the cybersecurity measures laid down in the same decree.^55^ Pursuant to Article 3 of DPCM 2, the cybersecurity incident notification carried out by NIS entities complies with the reporting obligations of Article 14 and 16 NISD, which require notification without undue delay.

If the entity becomes aware of new significant elements, including specific vulnerabilities exploited or—more generally—the detection of events in any way related to the incident, the notification shall be amended without undue delay from the moment of awareness, unless a prosecuting judicial authority has previously requested specific needs of investigation secrecy.^56^ Moreover, upon request of the Italian CSIRT, the entity who notified an incident shall, within six hours of the request, update the notification—with the exception of a case with specific needs of investigation secrecy.^57^

Article 4 DPCM 2 foresees further voluntary incident reporting for entities that are encompassed by the Perimeter. The CSIRT must give priority to mandatory notifications, before it deals with voluntary notifications. These notifications concern (a) incidents, related to ICT assets, which are not covered by Annex A; and (b) incidents, covered by Annex A, relating to entities’ networks, information systems and computer services not included in the list of identified ICT assets. To date, the Italian law does not require notification of cybersecurity threats as foreseen in the NIS2 Proposal.

With regard to cybersecurity measures, Annex B of DPCM 2 contains a complex and highly detailed taxonomy of cybersecurity measures. These measures under the heading of technical controls which are grouped according to their functions, i.e. identify, protect, detect, respond, and recover, are divided into two categories. The measures under category “A” of appendix no. 1 to Annex B must be applied to the ICT assets within six months from the date of transmission of the lists of ICT goods, or, if transmission took place before the date of entry into force of DPCM 2, within six months from the latter date; deadlines are extended up to thirty months for the measures falling under category “B”.^58^ Annex B of DPCM 2 accounts for 21 technical controls and 51 sub-controls in total. Entities shall notify the Cybersecurity Agency without undue delay of the adoption of such measures;^59^notification is also required for relevant updates.^60^

Interestingly, DPCM 2 specifically provides for information security related aspects. Annex C identifies several baseline cybersecurity controls that apply to the list of subjects in the Perimeter, the lists of the description of the architecture and components, as well as the risk analysis, elements of the incident notification reports, and the documentation related to the cybersecurity measures referred to in Annex B.^61^ Pursuant to Article 9(2) DPCM 2, these measures shall be applied within sixty days from the entry into force of DPCM 2.

The vast array of measures foreseen in Annex B and C of DPCM 2 largely correspond to security requirements under the NIS2 Proposal. For example, supply chain cybersecurity risk management (Article 18[2][d] and 18[3] NIS2 Proposal) corresponds to the control no. 2.5 of Annex B. In sum, the cybersecurity risk management measures and incident notification provisions of the Perimeter with the exception of notification timeframe are very similar to that in the NIS2 Proposal. As mentioned in the previous section, Article 1(5) Decree Perimeter lays down the legal basis for updating DPCM 2 at least every two years. The flexible national legislation, built around governmental decrees, avoids an overly prescriptive normative framework as it can be easily and (relatively) rapidly amended.

### Supervision: the role of the new national cybersecurity agency

The *Decreto-Legge* 14 June 2021 no. 82 (hereinafter, DL 82) established the national Cybersecurity Agency with a view to taking over the role of the national cybersecurity authority^62^ as a single point of contact for the purposes of the NISD^63^ and the national cybersecurity certification authority for the purposes of the CSA^64^. Therefore, inspection and audit activities, once entrusted to the Ministry of Economic Development and the Presidency of the Council of Ministers,^65^ now fall under the Agency’s competence. In that regard, chapter IV of DPR 54 stipulates supervisory powers and procedures vis-à-vis inspections and audits in relation to compliance with the various obligations imposed by the implementing decrees of the Perimeter.^66^ Besides regular monitoring based on the agenda of the Agency, chapter IV also foresees ad hoc inspections if deemed necessary in exceptional cases (e.g. as a direct result of incident notifications, non-compliance with any of the obligations resulting from the application of the relevant legislation and notifications from other public authorities).^67^ Audit activities are carried out through analysis and documentary checks, in order to ascertain compliance with the Perimeter Decree and its implementing decrees.^68^ Article 16(5) and 16(6) DPR 54 sets forth deadlines for the conclusions of different types of inspections guaranteeing timely completion of the procedures.^69^ Notwithstanding the higher administrative burden that may arise for IEs under the NIS2 Proposal, the comprehensive Italian provisions on supervision have already set the ground for compliance with the relevant NIS2 Proposal measures and will only require minor adaptions.

As the Commission estimated an increase in costs of 20/30% for national competent authorities with the adoption of the NIS2 Proposal^70^, Article 18(1) DL 82 already foresees an increase of the Agency’s budget allocation from €2 M in 2021 to €122M in 2026.^71^ Whether the budget will suffice to cover potential new tasks assigned to the Agency under the NIS2 Proposal and its increased scope of application as well as an increased threat level remains to be seen.

### Enforcement and sanctions

The Decree Perimeter introduces a range of different administrative sanctions for failing to meet the obligations imposed by the Decree Perimeter and its implementing decrees. For example, non-compliance with the duty to draw up, update and submit the lists of networks, information systems and IT services is subject to an administrative fine that ranges from €200,000 to €1.2 M, whilst failure to notify cybersecurity incidents or implement cybersecurity measures face fines in the range of €250,000–€1.5 M. Interestingly, more severe sanctions are imposed for non-compliance with procurement requirements: an entity that fails to notify the supply contract of ICT assets to the CVCN and does not comply with the conditions laid down by the CVCN can be fined up to €1.8 M.^72^

Article 1(10) Decree Perimeter—similarly to Article 29(5)(b) NIS2 Proposal—provides for the application of an accessory administrative sanction in the form of a temporary ban of three years against any person discharging managerial responsibilities at administrative or control level in the entity concerned. Further, Article 1(11) Decree Perimeter also foresees a criminal sanction of imprisonment of one to three years for the provision of false information, data or factual elements, or omission to communicate the aforementioned data, in order to hinder or influence the completion of the procedures related to incident notification, cybersecurity management measures, procurement or inspection as well as supervision activities.

## The German cybersecurity legal framework

German cybersecurity regulation precedes the NISD with the Act on improving the security of information technology systems (ITSiG) of 17 July 2015^73^, and the Regulation for Determining Critical Infrastructures pursuant to the BSI Act (BSI-KritisV) of 22 April 2016^74^ (Fig. 1). The entry into force of the NISD in 2016 only required subsequent minor changes by the first Regulation to change the BSI-KritisV (1st BSI KritisVÄndV) of 21 June 2017^75^ and the Act to implement the NISD of 23 June 2017^76^ in order to comply with EU law.

On 18 May 2021, the German parliament hastily passed the ITSiG 2.0^77^ at the end of the 19th legislative period parliament (Fig. 1).^78^ The ITSiG 2.0 responds to persisting unsolved issues of IT security in the field of critical infrastructures and beyond by adapting and advancing protection measures and defence strategies. 79 The Act primarily foresees changes and amendments to the central German cybersecurity act, the Act on the Federal Office for Information Security (BSIG)^80^. This includes regulations on the use of so-called critical components and the new category of companies of special public interest. Further, the mandate of the German regulatory authority for IT security, the Federal Office for Information Security (*Bundesamt für Sicherheit in der Informationstechnik* [BSI]) is expanded and strengthened. Notably, the ITSiG 2.0 is complemented by a new Regulation on Critical Infrastructures (2nd BSI KritisVÄndV)^81^ which entered into force on 1 January 2022 and amended several sectors by introducing new critical infrastructure types (Fig. 1). At the same time, thresholds for existing infrastructures are lowered, meaning that more infrastructures are encompassed as critical. Finally, the ITSiG 2.0 also changes and amends the Telecommunications Act (TKG)^82^, the Energy Economy Act (EnWG)^83^, the Foreign Trade and Payments Ordinance (AWV)^84^, the Social Code X (SGB X)^85^ and a variety of *lex specialis* that regulate critical sectors outside the scope of the BSI Act.

### The scope of German cybersecurity legislation: the BSIG amended by ITSiG 2.0

The ITSiG 1.0 already incorporated most parts of the NISD and thus, only minor changes where required by the NISD implementing act. The amendments included rules on digital service providers^86^, a section on the restoration of the security of functionalities of information technology systems in outstanding cases^87^, as well as regulations on information sharing and cooperation with the military counterintelligence service and the federal intelligence service^88^. The 1st BSI KritisVÄndV of 2017 then introduced the sectors finance and insurance, transport and traffic as well as health to the list of critical sectors, while only requiring minor amendments and clarifications with regard to the determination of critical infrastructures in the field of energy, water, food and information communication technologies in order to fully comply with the standard of the NISD.

The ITSiG 2.0 of 2021 expands the scope of application of the central cybersecurity law, the BSIG, to further new sectors: municipal waste with essential service municipal waste disposal (collection, disposal, recycling), and special public interest entities (SPIE). Similar to the NIS2 Proposal distinction of EEs and IEs, the SPIEs are distinguished from category of critical infrastructure since there importance does not pass the threshold of criticality, but they are nevertheless considered worth of protection. The SPIE category includes entities producing or developing goods encompassed by § 60 I No. 1 and 3 AWV (defense, arms, federal IT)^89^, entities of particular economic importance due to their size (economically relevant entities)^90^ and entities that utilize hazardous materials within their operational area (chemicals)^91^. The German legislator thus already employs the distinction foreseen by the NIS2 Proposal by distinguishing between ‘critical’ (i.e. essential) entities and ‘important’ entities. However, the entities considered as ‘important’ do not correspond to the IEs under the NIS2 Proposal, but are a unique feature of German law.

The primary subject of the new regulations remains operators of critical infrastructures (CRITIS). They have an obligation to register the critical infrastructure with the BSI,^92^ meaning that they have to self-identify themselves as CRITIS operators. In this respect, the German approach corresponds to the one foreseen by Article 25 NIS2 Proposal with a registry for ‘EE’ and ‘IE’ maintained by ENISA.^93^ As regards the scope of application, micro-enterprises are excluded from the scope of the BSIG. The BSI-KritisV determines quantitative thresholds for the entities encompassed, above which they will be considered a CRITIS operator. The obligation to register allows Germany to comply with the requirement to identify OESs under the NISD.

### Cybersecurity risk management and reporting obligations

As regards cybersecurity risk management, CRITIS operators have to implement appropriate organisational and technical measures. Sector-specific security standards can be approved by the BSI as amounting to appropriate measures,^94^ providing legal certainty for the entities concerned in terms of compliance. ITSiG 2.0 introduced the obligation to operate state of the art attack detection systems from 1 May 2023 onwards.^95^ In order to support this, the BSI provides a Malware Information Sharing Platform (MISP).^96^ The determination of a specific cybersecurity measures is rather unique and has also been criticised since there is widespread consensus among German scholars that laws should refrain from detailing technical protection measures.^97^ With the entry into force of the CSA, also certification of security products and services gains importance. Complementing the certification procedures of the CSA, § 9c BSIG introduces a voluntary IT security label to improve consumer information. In line with the EU legal framework on cybersecurity certification, the label is entirely voluntary to guarantee market access for EU competitors.

§ 8a III BSIG now also foresees a biannual obligation to prove compliance with the obligation to implement security measures. In the case of a significant disruption, entities are obliged to disclose the information necessary to handle the disruption to the BSI upon request.^98^

Similar to the Italian approach and the NIS2 Proposal, new IT security obligations include inter alia supply chain security, meaning that suppliers, i.e. manufacturers of critical components, will be subject to certain obligations to safeguard the supply chain. This includes an obligation to notify planned first-time use of a critical component to the Federal Ministry of the Interior under § 9b I BSIG. According to § 9b II BSIG, critical components must not be put into use before the expiry of a two-month review period. The notification must include a declaration on the trustworthiness of the manufacturer. In this declaration the manufacturer must provide information on its organisational structure and how it ensures that the component does not have technical features that specifically allow misuse, in particular for the purpose of sabotage, espionage or terrorism with regard to the security, confidentiality, integrity, availability or functioning of the CRITIS.^99^ Critical components are IT products that are used in critical infrastructures, for which disruptions of their availability, integrity, authenticity and confidentiality may lead to a failure or significant impairment of the functionality of CRITIS or to threats to public safety, and which are either designated as critical components by law or realise a critical function.^100^ The use of critical components can be prohibited if the supplier is not considered trustworthy.^101^ Accordingly, supply chain security focuses on the risks associated with foreign technological presence in the Union corresponding to the cybersecurity framework applicable to 5G networks and replicates the ratio of the EU coordinated supply chain risk assessments foreseen by Article 19 NIS2 Proposal.

§ 8b IV BSIG requires CRITIS operators to report without undue delay disruptions of the availability, integrity, authenticity and confidentiality of information technology systems, components or processes, which have resulted in a failure or have a significant impact on the functioning of the critical infrastructures operated by the entity concerned. In line with the NIS2 Proposal, this obligation also includes disruptions that have the potential to result in a failure or may have a significant impact on the service functioning. This extensive reporting obligation is not novel but has already been part of ITSiG 1.0. In contrast, pursuant to § 8c III BSIG, DSPs are only obliged to notify incidents that have a substantial impact on the service that they provide.

### Supervision: strengthening the BSI

A central instrument of the ITSiG 2.0 is the security of communication technologies of the Federal Administration, for which responsibility lies with the BSI. The BSI is conferred powers of control and information with regard to technology, strategy, planning and regulations.^102^ The BSI is also empowered to process protocol data including the recording of data concerning technical events or conditions within IT systems of the Federal Administration in order to detect malware.^103^

Further, the mandate of the BSI is strengthened and extended in a variety of fields. In that regard, the ITSiG 2.0, inter alia, sets out the tasks and powers of BSI as the national cybersecurity authority within the meaning of Article 58 CSA (certification). In addition, under § 3 I 2 No. 14a BSIG, the BSI gains competence as regards consumer protection and consumer information in the area of IT security. As mentioned above, the ITSiG 2.0 also specifies the BSI’s task of developing requirements and recommendations together with conformity testing and confirmation for IT products.^104^ As regards threat intelligence, § 7b IV BSIG now authorises the BSI to actively conduct port-scans and operate honeypots. Similar as foreseen by the NIS2 Proposal, the BSI—in its role as the competent NIS authority—gains competence to issue orders in the telecoms sectors, such as orders against telecommunications and telemedia providers to avert specific threats to IT security.^105^

### Enforcement and sanctions

Along with the strengthening of supervisory powers and the extended scope of German cybersecurity legislations goes stronger enforcement. The catalogue of offences in § 14 BSIG is extended by encompassing a wide variety of offences including failure to register as a CRITIS operator and unsolicited use of IT security marks. Almost all material and procedural obligations of the BSI are now subject to a sanctioning regime in case of non-compliance. The maximum administrative fine applicable is increased to €2 M.^106^

## Conclusion

Harmonised cybersecurity rules at EU level are the most efficient way to increase the level of cyber resilience [2]. Isolated moves forwarded by Member States contravene the rationale of a more coherent level playing field across the EU. Thus, striving for more cyber resilience necessarily requires a coordinated approach by Member States to avoid fragmentation. At national level, legal regulations with a predictable short life-span and a highly fragmented micro-level regulatory framework (as in Italy with two Decrees-Law, four Prime Ministerial Decrees, a Presidential Decree and a series of acts, communications and determinations of various committees) represent a challenge not only in terms of coherence, but also in terms of compliance for the entities concerned.

With the fast handling of the NIS2 legislative process, which necessarily hinges on a large consensus among the three co-legislators, political agreement has already been reached between the co-legislators in May 2022.^107^

The analysis above shows an overall high level of maturity of the recently adopted Italian and German cybersecurity laws against the background of selected regulatory drivers of the NIS2 Proposal.

Having regard to the significant extension of the scope of application of a NIS2 Directive and the identification process, the German approach seems, at first glance, almost aligned with the NIS2 legal standard. In compliance with the NIS2 approach, CRITIS have to self-identify themselves as critical infrastructures. Hence, the German approach duplicates the NIS2 standard as it already encompasses, for instance, the waste management sector. Also, the inclusion of so-called SPIEs partly corresponds to the enlarged scope of the NIS2 Proposal but requires amendments with regard to, inter alia, postal and courier services, chemicals, food production, processing and distribution. Conversely, the Italian Decree Perimeter establishes that the entities that fall under the scope of the Perimeter shall be identified by the competent public administrations. As regards the scope of application, the Perimeter covers all public administrations, including interior and defence, enhancing and extending therefore the scope of the NISD to uphold national security. Further sectors include social security and labour, thereby addressing social stability as essential for the functioning of the Italian state. Obviously, both Member States must adapt their frameworks to the new distinction between IEs and EEs, although both national regimes already differentiate between different levels of importance.

In terms of cybersecurity management measures and reporting obligations, the two national cybersecurity legislations correspond, in general, with the provisions of the NIS2 Proposal. For example, both Member States require cybersecurity risk management of the supply chain. In that regard, the German cybersecurity legislation introduces a trustworthiness assessment of the manufacturer that mirrors the EU coordinated risk assessment of critical supply chains of Article 19 NIS2 Proposal, which would potentially be rendered obsolete in light of the new EU level procedure. Minor adjustments relate to the notification timeframes, which under the NIS2 Proposal will be aligned with a uniform notification procedure.

As regards the role of the supervisory agencies, Italian and German legislators deemed appropriate to strengthen and extend the mandate of the *Agenzia per la Cybersicurezza Nazionale*—established in 2021—and the *Bundesamt für Sicherheit in der Informationstechnik, *respectively. In both Member States, the cybersecurity agency will be the national competent authority and singular point of contact for the purposes of the NISD and national cybersecurity certification authority for the purposes of the CSA. Both agencies are empowered to conduct audits and tests on ICT products for IT security purposes.

Finally, both national legal frameworks provide for a range of different and severe administrative fines for failing to meet the obligations laid down in the relevant national laws. In that respect, the GDPR-aligned sanctioning model of the NIS2 Proposal (i.e. fines up to €10 M or 2% of the total annual worldwide turnover, whichever is higher) is not yet reflected in the national legal frameworks.

To conclude, the assessment of the national legal frameworks against the NIS2 Proposal shows in line with Bitkom [1] that bringing national regulations in motion in the run-up to new European legislation requires subsequent adjustments, which could have been avoided. This creates unnecessary burden for the entities concerned, which may have to adapt their policies anew. More importantly, efforts of national legislators may prove gratuitous. For instance, during the legislative process for the ITSiG 2.0, the NIS2 Proposal had already been published and the German legislator must have been aware of the speedy nature of the legislative process at EU level^108^. There was room for manoeuvre, i.e. adapting the national legislation to the Proposal ahead of the trilogue negotiations, if the legislator insisted on passing a law ahead of the Council vote under the French Presidency. Advancing with ITSiG 2.0 ahead of a vote on a NIS2 Directive means that entities in Germany will face an ‘avoidable’ ITSiG 3.0 in the near future with the consequence of adapting business policies and cybersecurity action plans.

The complexity that will necessarily arise from the transposition of the NIS2 Directive may be a “blessing in disguise” in that Member States may rework their national cybersecurity legislation that may be fragmented. Legislators should seize the opportunity to harmonise their national cybersecurity legislation within a single, organic, comprehensive and coherent legislative text reaching the objectives provided for by the NIS2 Directive and, at the same time, taking into account specific national demands. This will greatly benefit national competent authorities, market operators and legal professionals and would avoid overlaps and duplicative requirements under different legal acts [22].

With the COVID-19 pandemic accelerating digital transformation of the Single Market, the European Commission also speeded up the review of the first piece of EU-wide cybersecurity legislation, the NIS Directive^109^. Originally foreseen for May 2021, the Commission presented the review as early as December 2020 together with a Proposal for a NIS2 Directive (European Commission 2020b). Almost in parallel, some Member States strengthened (or adopted) national laws beyond the scope of the NIS Directive to respond adequately to the fast-paced digital threat landscape. Against this backdrop, the article investigates the national interventions in the field of cybersecurity recently adopted by Italy and Germany. In order to identify similarities and divergences of the Italian and German national frameworks with the European Commission’s Proposal for a NIS2 Directive, the analysis will focus on selected aspects extrapolated from the Commission Proposal, namely: i) the enlarged scope; ii) detailed cybersecurity risk-management measures; iii) more stringent supervisory measures; and, iv) stricter enforcement requirements, including harmonised sanctions across the EU. The article concludes that the national cybersecurity legal frameworks under scrutiny already match the core of the proposed changes envisaged by the NIS2 Proposal.

## Footnotes

1 Whereas the 2020 threat landscape combined 22 different reports (seven ‘strategic’ reports and 15 more technical analysis on top cyber threats), ENISA [8] discusses the first eight cybersecurity threat categories in terms of impact to shed light on the major change from the 2018 threat landscape as the COVID-19-led transformation of the digital environment.

2 Note: The Proposal for a CER Directive would benefit from further clarification, in particular, having regard to the definition of ‘resilience’: it is unclear whether the current scope of the CER “points specifically only to physical (non-cyber) aspects of resilience or not”, cf. DIGITALEUROPE [7]. Overlaps and duplicative requirements with a NIS2 Directive should be avoided.

3 Relevant amendments of the Parliament and Council drafts in their role as co-legislators will be addressed in the footnotes.

4 Directive (EU) 2016/1148 of the European Parliament and of the Council of 6 July 2016 concerning measures for a high common level of security of network and information systems across the Union, OJ L 194, 19.07.2016, 1–30.

5 For an overview of the systematic and structural changes see Schmitz-Berndt [19], Sievers [20], Brighi and Chiara [3] and Gruber and Ségur-Cabanac [17].

6 Art. 2(1) NIS2 Proposal.

7 The Council proposes to exclude public administrations.

8 Art. 2(1) NIS2 Proposal.

9 As regards the relevance of the manufacturing sector see Chiara [6].

10 Recital 7; Art. 2(1) NIS2 Proposal.

11 Art. 2(1), (2) NIS2 Proposal.

12 Art. 18 NIS2 Proposal.

13 The European Parliament proposed a compromise in its position whereby incidents that significantly disrupt the availability of the service provided are to be reported within 24 h; incidents that have a significant impact on the entity other than on the availability of the services should be reported within 72 h.

14 The European Parliament opposes this extension of mandatory reporting and favours a reporting obligation that is restricted to incidents that have actually resulted in harm. Reporting of incidents that only have the potential to result in harm shall be subject to voluntary notification.

15 European Parliament and Council support the voluntary reporting of cyber threats.

16 Cf. Recital 43 NIS2 Proposal.

17 Commission Recommendation (EU) 2019/534 of 26 March 2019 Cybersecurity of 5G networks, OJ L 88, 29.03.2019, 42.

18 Recital 47 NIS2 Proposal.

19 Recital 70, Arts. 29, 30 NIS2 Proposal.

20 Recital 60, Art. 17(1) NISD.

21 As regards the fine framework, the European Council as co-legislator proposed a differentiation between EEs and IEs with €4 M or 2% of annual turnover in the case of EEs and €2 M or 1% of annual turnover in the case of IEs, respectively.

22 Cf. Recital 76 NIS2 Proposal.

23 A translation of the Act into English is available at https://encavibs.uni.lu/wp-content/uploads/sites/158/2022/01/ITALY_decreto_NIS_EN.pdf (accessed 09 June 2022).

24 Certain amendments were made to this act by Decree Law No. 162 of 2019, in terms of extended time limits and other provisions on public administration. Moreover, the Decree-Law no. 105 has been converted into law on 28 February 2020 (*Legge no. 8 del 28 febbraio 2020*).

25 Cf. Article 1(1) Decree Perimeter.

26 Art. 1(8)(a) Decree Perimeter. Thus, Art. 1(8)(b) Decree Perimeter mandates that the notification duties laid down by the Perimeter constitute compliance with Arts. 14 and 16 NISD.

27 *Decreto del Presidente del Consiglio dei Ministri 30 luglio 2020, n. 131.*

28 These lists have to be updated annually.

29 *Decreto del Presidente del Consiglio dei Ministri 14 aprile 2021, n. 81.*

30 *Decreto del Presidente della Repubblica 5 febbraio 2021, n. 54.*

31 *Decreto del Presidente del Consiglio dei Ministri 15 giugno 2021.*

32 *Decreto-Legge 14 giugno 2021, n. 82.*

33 Art. 7(3) DL 82.

34 Regulation (EU) 2019/881 of the European Parliament and of the Council of 17 April 2019 on ENISA (the European Union Agency for Cybersecurity) and on information and communications technology cybersecurity certification and repealing Regulation (EU) No 526/2013 (Cybersecurity Act).

35 Art. 4(1) DPCM 1.

36 Cf. Art. 1(2)(b) Decree Perimeter.

37 Art. 3(1) DPCM 1.

38 Art. 4 DL 82.

39 Art. 3(2) DPCM 1.

40 *Ibid.*

41 Art. 1(2-bis) Decree Perimeter.

42 Brunella Bruno (n 4), 27–28.

43 Art. 3(1)(i) DPCM 1.

44 Regulation (EU) 2019/452 of the European Parliament and of the Council of 19 March 2019 establishing a framework for the screening of foreign direct investments into the Union, OJ L79I, 21.03.2019, 1–14.

45 Against this backdrop, in the opinion of the author, the Italian legislator should benefit from the approach adopted by Annex I section 8 NIS2 Proposal: within the EEs, the EU legislator lists the “digital infrastructure” subsector, which explicitly includes cloud computing services, content delivery network providers, internet exchange point providers, DNS service providers, TLD name registries, data centre service providers, content delivery network providers, trust service providers and public electronic communications networks.

46 Art. 7(1) DPCM 1.

47 Art. 7(2)(b) DPCM 1.

48 Art. 1(1)(n) DPCM 1 defines “architecture and component parts” as the set of implemented architectures and components used at network, data and software level, including deployment on cloud computing platforms, as well as the procedures and information flows for accessing, acquiring, transmitting, storing, processing and retrieving the data needed to perform the IT services.

49 Art. 8 DPCM 1.

50 Art. 9 DPCM 1.

51 Art. 1(1)(h) DPCM 2 defines “incident” as any event of an accidental or intentional nature which causes the malfunctioning, interruption, even partial, or improper use of networks, information systems or IT services.

52 Art. 2(1) DPCM 2.

53 Art. 3(1) DPCM 2.

54 Art. 3(4) DPCM 2.

55 *Ibidem*.

56 Art. 3(5) DPCM 2.

57 Art. 3(7) DPCM 2.

58 Art. 8(1) DPCM 2.

59 Art. 8(3) DPCM 2.

60 Art. 8(4) DPCM 2.

61 Art. 9(1) DPCM 2.

62 The Agency takes charge of the tasks—in the cybersecurity field—previously assigned to the Ministry of Economic Development, the Presidency of the Council of Ministers—but for the task of identifying the entities falling in the Perimeter pursuant to Art. 3 DPCM 1, and the Information Department for Security.

63 Art. 7(1)(d) DL 82.

64 Art. 7(1)(e) DL 82.

65 Art. 1(6)(c) Decree Perimeter.

66 According to Art. 14(1) DPR 54, the requirements upon which the Agency shall vigilate include i) drawing up, updating and transmitting of lists related to network, information systems and IT services; ii) cybersecurity incident notification duties to Italian CSIRT; iii) adoption of cybersecurity management measures; iv) notification to the national cybersecurity evaluation centre (CVCN) as regards procurement procedures; v) adoption and deployment of ICT assets which have passed the tests and conditions set by the CVCN; vi) cooperation in testing activities; vii) compliance with Agency and CVCN requirements.

67 Art. 16(1) DPR 54.

68 Art. 17(1) DPR 54.

69 Art. 16(5) DPR 54 foresees completion within 120 days from the date of notification, whereas Art. 16(6) DPR 54 foresees a time limit of 90 days for reviewing any evidence acquired during an audit, where such evidence presents elements worthy of further investigation, and/or analysis, detection, acquisition and verification of relevant factual and legal elements (see Art. 18(1) DPR 54).

70 European Commission, ‘Impact Assessment Report Accompanying the document Proposal for a Directive of the European Parliament and of the Council on measures for a high common level of cybersecurity across the Union, repealing Directive (EU) 2016/1148’ SWD(2020) 345 final, 83.

71 €2 million for the year 2021, €41 M for the year 2022, €70 M for the year 2023, €84 M for the year 2024, €100 M for the year 2025, €110 M for the year 2026 and €122 M for the year 2026.

72 Art. 1(9) Decree Perimeter.

73 Gesetz zur Erhöhung der Sicherheit informationstechnischer Systeme vom 17. Juli 2015, BGBl (German Federal Law Gazette) I p. 1324.

74 Verordnung zur Bestimmung Kritischer Infrastrukturen nach dem BSI-Gesetz vom 22. April 2016, BGBl. I, 958.

75 Erste Verordnung zur Änderung der BSI-Kritisverordnung vom 21. Juni 2017 (1. BSI KritisVÄndV), BGBl. I, 1903.

76 Gesetz zur Umsetzung der Richtlinie (EU) 2016/1148 des Europäischen Parlaments und des Rates vom 6. Juli 2016 über Maßnahmen zur Gewährleistung eines hohen gemeinsamen Sicherheitsniveaus von Netz- und Informationssystemen in der Union vom 23. Juni 2017, BGBl. I, 2017.

77 Zweites Gesetz zur Erhöhung der Sicherheit informationstechnischer Systeme vom 18. Mai 2021, BGBl I, 1122 and 4303.

78 The ITSiG 2.0 entered into force on 28 May 2021.

79 Cf. Deutscher Bundestag, BT-Drs. 19/26106, 1 et seq.

80 Gesetz über das Bundesamt für Sicherheit in der Informationstechnik vom 14 August 2009, BGBl. I, 2821.

81 Zweite Verordnung zur Änderung der BSI-KritisVvom 06. September 2021, BGBl. I, 4163.

82 Telekommunikationsgesetz vom 22. Juni 2004, BGBl. I p. 1190, replaced by Telekommunikationsgesetz vom 23 Juni 2021, BGBl. I, 1858.

83 Gesetz über die Elektrizitäts- und Gasversorgung (Energiewirtschaftsgesetz) vom 07. Juli 2005, BGBl. I, 1970 and 3621.

84 Außenwirtschaftsverordnung vom 02. August 2013, BGBl. I, 2865.

85 Zehntes Buch Sozialgesetzbuch vom 18 Januar 2001, BGBl. I, 130.

86 Now included inter alia as § 2 XI, XII, § 8c BSIG.

87 See § 5a BSIG.

88 See for instance § 3 I s. 2 no. 13 b, § 5 V s. 2 no. 2 & 3, VI s. 1 no. 3 & 4 BSIG.

89 See § 14 XIV no. 1 BSIG. These goods also fall under export control.

90 See § 14 XIV no. 2 BSIG.

91 See § 14 XIV no. 3 BSIG.

92 See § 8b III BSIG.

93 For the identification process see Axel Freiherr v.d Bussche and Tobias Schelinski in: Andreas Leupold, Silke Glossner, et al. (eds), Münchner Anwaltshandbuch IT-Recht (4th ed. 2021), Part. 7.1, marginal no. 20 et seq [16]

94 § 8a II BSIG.

95 See § 8a Ia BSIG.

96 https://misp.bsi.bund.de/users/login.

97 Cf. Gerrit Hornung, Das IT-Sicherheitsgesetz 2.0: Kompetenzauswuchs des BSI und neue Pflichten für Unternehmen, NJW [18], 1985, 1987.

98 § 8b IVa BSIG.

99 § 9b III BSIG.

100 § 2 XIII BSIG.

101 § 9b IV, V BSIG. According to Cerulus [5], this inter alia gives more power to the Ministry of the Interior to block contracts that for instance do not match the security policy goals of Germany, the EU and NATO.

102 § 4a BSIG.

103 § 5a BSIG.

104 § 9a I and II BSIG.

105 § 7c and d BSIG.

106 § 14 V BSIG.

107 The agreement has not been published yet as of time of writing this paper.

108 There was room for manoeuvre, i.e. adapting the national legislation to the Proposal ahead of the trilogue, if the legislator insisted on passing a law ahead of the Council vote on the Proposal.

109 Directive (EU) 2016/1148 of the European Parliament and of the Council of 6 July 2016 concerning measures for a high common level of security of network and information systems across the Union, OJ L 194, 19.07.2016, 1–30.
